# Improving nutritional composition index of seeds of common bean lines

**DOI:** 10.1371/journal.pone.0355162

**Published:** 2026-08-03

**Authors:** Juan Carlos Suárez, Paola Andrea Polanía-Hincapié, Vivian Yorlady Ramón-Triana, Jennifer Wilker, Idupulapati M. Rao

**Affiliations:** 1 Programa de Ingeniería Agroecológica, Facultad de Ingeniería, Universidad de la Amazonia, Florencia, Colombia; 2 Centro de Investigaciones Amazónicas CIMAZ Macagual César Augusto Estrada González, Grupo de Investigaciones Agroecosistemas y Conservación en Bosques Amazónicos-GAIA, Universidad de la Amazonia, Florencia, Colombia; 3 Programa de Maestría en Sistemas Sostenibles de Producción, Facultad de Ciencias Agropecuarias, Universidad de la Amazonia, Florencia, Colombia; 4 Programa de Doctorado en Ciencias Naturales y Desarrollo Sustentable, Facultad de Ciencias Agropecuarias, Universidad de la Amazonia, Florencia, Colombia; 5 Alliance of Bioversity International and CIAT, Cali, Colombia; Universidade Federal de Minas Gerais, BRAZIL

## Abstract

Common bean cultivars developed through interspecific crosses with five different *Phaseolus* species (*P. vulgaris*, *P. coccineus*, *P. acutifolius*, *P. dumosus*, and *P. parvifolius*) to combine desirable nutritional characteristics in their seeds are important for improving nutritional value and food security in the Global South. In this study, we evaluated the nutritional composition (ash, crude protein, fat, fiber, hemicellulose, cellulose and lignin) and mineral status (Fe, Zn, K, Mg and other minerals) of 112 common bean genotypes including bred lines that were developed from interspecific crosses and were grown under acidic soil and high-temperature stress conditions in Colombia’s Amazon region. Genotypes tested for nutritional composition and mineral status were grouped into three different typologies: high Fe and Zn content (HFZ); high dietary fiber and cellulose (HDFC); and low dietary fiber and cellulose (LDFC). Based on genotypic differences found and grouping of genotypes according to fiber content and mineral status, we developed nutritional composition index (NCI) as a selection criterion to assess nutritional value of common bean. The NCI was created by considering variables that have both positive and negative impacts on nutritional quality. Among the genotypes evaluated, bean materials with HFZ typology exhibited a mean NCI value of 0.53, including four genotypes (SEF 49, SEF 10, NCB 280, VAP 15) developed from interspecific crosses. Four genotypes of *P. acutifolius* exhibited HDFC typology with a mean NCI value higher than 0.3 compared to other genotypes. Among the genotypes of LDFC typology, *P. acutifolius* accession G 40001 exhibited the lowest NCI value. Regarding fiber content, HDFC typology included mainly genotypes of *P. acutifolius* species (G 40250, G 40253, G 40254) with NCI values higher than 0.3. NCI showed higher values of correlation coefficients with crude protein, K, Fe and Zn. Combining NCI and NQI values—which include bioactive compounds and antioxidant activity—helped identify common bean lines with superior nutritional quality. Results from this study highlight the superior nutritional value of SEF 49 and SEF 10 based on NCI values and these two genotypes could serve as valuable parents in future bean breeding programs aimed at improving the nutritional value of common bean.

## 1. Introduction

*Phaseolus* beans, the most widely distributed legume of the Fabaceae family [1], are fundamental to global nutrition due to their high protein and carbohydrate content [2]. They exhibit strong antidiabetic, cardioprotective, and anticancer properties [3], containing twice the protein of cereals and being rich in fiber, vitamins, and minerals [4,5]. They are especially important for low-income populations in Latin America and Africa [6]. Facing the global malnutrition crisis affecting 149 million children according to World Health Organization [7], beans represent a nutritious, affordable, and sustainable food solution [8].

Phaseolus beans adapt to tropical, subtropical and temperate environments [1,9,10]. This environmental adaptation creates biodiversity and genetic variation, producing diverse landraces [11]. While yield improvement has been prioritized, less focus has been given to enhancing nutritional qualities like protein, minerals and fats [12]. Interspecific crosses are fundamental for improving nutritional traits because they allow the incorporation and combination of valuable nutritional characteristics from different *Phaseolus* species (*P. vulgaris, P. coccineus, P. acutifolius, P. dumosus*), thus broadening the genetic base available for breeding. This is particularly important given that modern varieties have reduction in key micronutrient levels during domestication [12], and interspecific crosses allow the recovery of these valuable nutritional characteristics from wild or less domesticated species [13]. The effectiveness of this strategy has been demonstrated with successful examples such as the SEF lines (derived from *P. vulgaris × P. coccineus × P. acutifolius* crosses) with high iron content and VAP 15 line (*P. vulgaris × P. acutifolius × P. parvifolius*) with high protein and zinc content. This approach is especially relevant for improving food security in developing countries, as it enables the development of varieties with better nutritional value, compensating for the historical focus of breeding programs that have mainly centered on yield, neglecting nutritional and quality aspects. Therefore, it is necessary to further investigate nutritional characteristics of bean seeds that are important for human nutrition to identify advanced common bean lines that can combine stress tolerance with high nutritional composition of seeds [14].

Studies on bean varieties have revealed diverse nutritional profiles. Greek local varieties showed protein contents of 18.8–23.9%, fiber 7.77–12%, and fat 1.84–2.58% [1]. Chilean varieties presented protein between 20.3–30.3%, iron 48.6–109 mg kg^−1^, and zinc 23–52.6 mg kg^−1^ [15]. Among species, *Phaseolus vulgaris* showed higher protein content (20.4%) than *P. lunatus* (19.6%) and *P. coccineus* (17.2%), as well as higher levels of fat and fiber [16]. Brazilian varieties reported carbohydrates of 60–66 g/100g DM and dietary fiber of 21–24 g 100 g^−1^ DM [17]. With the objective of finding advanced lines of common bean and other genotypes of *Phaseolus* species that can adapt to acid soil and high temperature stress conditions [18], genotypes with higher values of seed yield have been identified [19,20]. In addition to having good agronomic performance, these genotypes have been shown to have a high content of bioactive compounds and antioxidant activity that are very important for human health [21]. Among these genotypes, we highlighted BFS 10, GGR 147, SMG 12 and SMG 21 which are advanced common bean lines with grain yield values greater than 1,800 kg ha^−1^ [20,22] as well as three interspecific improved lines (SER 212, SER 213 and RRA 81), with nutritional quality index (NQI) values higher than 0.8 [21]. However, this same study found that some genotypes from *P. vulgaris* (SMG 20, INB 604, SMG 6) and *P. acutifolius* (G 40001, G 40177E3, G 40179) have very low NQI values.

Therefore, to better understand and characterize these genotypes’ nutritional properties for the development of nutritionally superior common bean varieties, it is necessary to conduct seed evaluations that include both bioactive compounds and antioxidant activity (reflected in the NQI [21]) as reported before, together with nutritional composition values in terms of fiber and minerals that could also be integrated into another index as nutritional composition index (NCI). Similar to NQI, NCI values could also operate on a scale of 0–1 and could be based on the same mathematical principle of variable transformation. The NQI evaluates components such as total phenolic content, total flavonoids, antioxidant activity, carotenoids, reducing sugars, and monomeric anthocyanins as positive variables, although condensed tannins were considered negative factors. [21]. It has been documented that *Phaseolus* spp. constitutes a significant source of secondary metabolites—including phenols, flavonoids, anthocyanins, and tannins—which classifies these as a functional food due to their capacity to neutralize reactive oxygen species, inhibit the initiation and progression of oxidative processes, and consequently contribute to the prevention of diseases associated with oxidative stress. These effects are positively correlated with the concentration of antioxidant-related compounds, as assessed by using assays such as 2,2′-azino-bis(3-ethylbenzothiazoline-6-sulfonic acid) (ABTS^•+^) radical cation-based assays for radical scavenging capacity, and ferric reducing antioxidant power (FRAP). On the other hand, the nutritional composition index (NCI) could include cellulose, soluble dietary fiber, various minerals (Mn, Mg, Cu, K, Fe, Ca, Zn), crude protein, crude fiber, hemicellulose, and lignin as positive variables, while sodium could be considered as a negative variable. This comprehensive seed evaluation, combining NCI and NQI values provides a complete tool for assessing and improving the overall nutritional quality of bean genotypes. Therefore, the main objective of this study was to characterize the fiber and mineral composition and develop NCI values based on different typologies for identifying nutritionally superior improved lines of common beans developed from interspecific crosses through breeding. We tested the hypothesis that significant variability in nutritional composition exists among different Phaseolus species, allowing for classification into distinct typologies. We also investigated the possibility of identifying nutritionally superior genotypes by using NCI values together with NQI values.

## 2. Materials and methods

### 2.1. Plant material

The nutritional characteristics of seeds harvested from a set of 112 genotypes including bred lines resulting from different crosses of *P. vulgaris*, *P. coccineus*, *P. dumosus*, *P. acutifolius* and *P. parvifolius* species were determined whose evaluated interspecific lines come from embryo rescue as well as crosses between different materials [23] (Table 1). The seeds used in this study were obtained from three field experiments conducted by Suárez et al. [20,22,24] at the Macagual Research Center of the University of the Amazon (1°37′ N, 75°36′ W), Colombia. The breeding lines were developed and provided by the Bean Program of the Alliance of Bioversity International and CIAT, and the germplasm accessions of *P. acutifolius* were provided by the “Seeds of the Future” germplasm bank and these are preserved under the treaty for genetic resources of the Food and Agriculture Organization of the United Nations under an Standard Material Transfer Agreement (SMTA). The previous studies evaluated, under combined acidic soil and high temperature conditions in the Colombian Amazon region, the morpho‑physiological, phenological, and agronomic differences among advanced lines and related species of *Phaseolus* (Mesoamerican and Andean common bean, tepary bean and its relatives). These studies were focused in identifying adapted genotypes and the physiological mechanisms (energy use, leaf cooling, partitioning of photosynthates to pod set and grain filling) that contribute to improved adaptation and yield, without any type of appraisal of the nutritional content, which is the object of the present study.

**Table 1:** Genotypes tested for the analysis of nutritional composition of seeds. Seeds were harvested from the plants grown under the acid soil and high temperature stress conditions of the Colombian Amazon.

Species or cross (number of genotypes)	Breeding line or germplasm accession
**Germplasm accession**	
*Phaseolus acutifolius* (n = 22)	G 40001, G 40058, G 40091, G 40177E3, G 40179, G 40192, G 40198, G 40203, G 40208, G 40210A, G 40221, G 40222, G 40236, G 40248, G 40248A, G 40249, G 40250, G 40253, G 40254, G 40261, G 40270, G 40276
**Breeding line**	
*Phaseolus vulgaris* (n = 46)	*Andean:* CALIMA [25], DAA 129, DAB 295 [26], ICA QUIMBAYA [27], SAB 618, SAB 686 [28]. *Mesoamerican:* AMADEUS [29], BFS 10, BFS 35 [30], DOR 390 [31], EMP 509 [32], GGR 131, NCB 280 [33], SCR 23, SCR 40, SCR 56, SCR 61 [30], SEN 135, SEN 136, SEN 46, SEN 48, SEN 52, SEN 70, SEN 97, SER 125, SER 16, SER 271, SER 316, SER 323, SER 48, SMN 65, SMN 68, SMN 99, SMR 101, SMR 133, SMR 138, SMR 139, SMR 155, SMR 156, SMR 173, SMR 180, SMR 39, SMR 43, SXB 412 [33], TIO CANELA [34], VAX 1 [35].
*P. vulgaris* × *P. acutifolius* (n = 5)	INB 604, INB 841 [36], SER 212, SER 213 [33], SIN 461−1 [37]
*P. vulgaris* × *P. acutifolius* × *P. parvifolius* (n = 1)	VAP 15 [38]
*P. vulgaris* × *P. coccineus* (n = 19)	*Andean:* RRA 101, RRA 123, RRA 13, RRA 177, RRA 57, RRA 60, RRA 68, RRA 69, RRA 79, RRA 81 [39] *Mesoamerican:* ALB 121, ALB 191, ALB 210, ALB 213, ALB 60, ALB 352, ALB 353 [40], BFS 142, BFS 81 [30].
*P. vulgaris* × *P. acutifolius* × *P. coccineus* (n = 13)	SEF 10, SEF 12, SEF 14, SEF 16, SEF 27, SEF 40, SEF 42, SEF 49, SEF 56, SEF 59, SEF 70, SEF 71, SEF 73 [41]
*P. vulgaris* × *P. acutifolius* × *P. coccineus* × *P. dumosus* (n = 5)	GGR 150, SMC 205, SMG 12, SMG 20, SMG 6 [33]
*P. vulgaris* × *P. dumosus* (n = 1)	SMR 84 [33]

During the crop growing season of the field experiments, the average recorded conditions were mean maximum temperature of 31.8 ± 0.8 °C, mean minimum temperature of 21.7 ± 0.6 °C, and mean total precipitation of 765 ± 54 mm. These environmental conditions did not vary significantly across the different seasons in which the evaluations of adaptation to acidic soils and high temperatures were conducted. The first experiment was established under an alpha-lattice design with 6x6 superblocks, structured in rows and columns with four replications. Each block contained 30 experimental genotypes, and one control (variety CALIMA) replicated six times (36 plots per block); a design selected to control spatial heterogeneity within the experimental site. The second and third experiments were implemented under a completely randomized block design with three replications, where each genotype constituted a random plot within the block. In all three experiments, each experimental unit consisted of three rows of 2 m length, with 0.6 m between rows and 0.15 m between plants, establishing a population density of 11 plants m^-2^. At harvest time, destructive sampling was performed in the central part of each plot, selecting a 1-m row segment containing five plants. The grains obtained from pod threshing were cleaned and dried at room temperature until they reached 10% moisture content. Subsequently, the samples were stored at 10°C until corresponding analyses were performed. For analysis, the seeds collected in these three field experiments were homogenized to obtain three samples (n = 3), each of which was analyzed for nutritional composition in triplicate in the laboratory.

These field experiments were carried out under acidic soil and high temperature stress, and humid conditions, following manual soil preparation procedures without the application of soil amendments or pest and disease control. The evaluated materials included some varieties and breeding lines such as ICA QUIMBAYA, AMADEUS, CALIMA, BFS 10, VAX 1, among others, which were repeated in at least two of the three experiments.

Among the materials evaluated, there are different advanced bean lines with the ability to adapt to drought conditions, and low-fertility acid soil stress (with low phosphorus and organic matter content and high acidity), such as ALB (small red kidney, black kidney) and BFS (small red). Also, Andean lines with capacity to adapt to drought conditions and with commercial traits at seed level such as color and size included DAA (fuchsia-red) and DAB (speckled-red, red-pink). The INB line evaluated exhibits resistance to common bacterial blight resulting from the interspecific cross between the tepary bean and common bean, while the RRA line exhibits resistance to root rot caused by *Pythium* and *Sclerotium* and the SCR line (red) and the commercial variety DOR 390 (black) possess recessive genes (bgm-1 [42]) for resistance to bean golden yellow mosaic virus. Different lines with heat and/or drought tolerance such as SAB (medium-sized cream and red mottled seeds), SEF (red), SER (red), SEN (black), SXB (cream) and SIN (brown) were evaluated. Lines with high mineral content (Fe and Zn) included the SMC (brown), SMG (pink), SMN (black) and SMR (red) lines. The VAP line evaluated was derived from the cross of *P. vulgaris × P. acutifolius × P. parvifolius* with heat tolerance; and the VAX-1 line (mottled-cream), was sensitive to Al toxicity. Three commercial varieties were used in the evaluation and these included ICA QUIMBAYA (red), CALIMA (mottled red), and AMADEUS (bright light red).

### 2.2. Sample treatment

For each bean material, 50 g of previously dehydrated seed (dry weight) was selected. Subsequently, the seed was ground to a fine powder with a particle size of 0.45 mm using a Grinder w8810 series mill (Beijing Kunlun Tech Co. Ltd., Beijing, China) and stored in an airtight container until further analysis. Three samples from each genotype in each experiment were analyzed to determine nutritional characteristics.

### 2.3. Nutritional composition

Proximate analysis of bean samples was performed according to the methods established by the Association of Official Analytical Chemists [43]. Crude protein (CP) content was calculated from the determination of nitrogen by the Kjeldahl method (DK 6 and UDK 139 VELP Scientifica, Italy) (AOAC Method No. 991.20) using the conversion factor of 6.25 [44]. Total fat content was determined according to the Randall method using petroleum ether (AOAC Method No. 991.36), in a semi-automatic solvent extractor (SER 148 VELP Scientifica, Italy). Ash content was determined by converting the organic matter into ash in a muffle furnace (1100°C, 22.9A Fisher Scientific, Spain) at 550 ± 5 °C until the sample was free of carbon, cooled in a desiccator, and the amount of ash was calculated (AOAC Method No. 930.30). Insoluble dietary fiber and crude fiber were determined according to the Weende method proposed by Van Soest et al. [45], using the fiber analyzer (FIWE 6 VELP Scientifica, Italy) (AOAC Method No. 962.09).

Atomic absorption spectroscopy (AA-7000, Shimadzu Co., Kyoto, Japan) was used to determine the mineral composition of calcium (Ca), magnesium (Mg), sodium (Na), potassium (K), copper (Cu), iron (Fe), zinc (Zn) and manganese (Mn) with the acid digestion method [46]. Verification of the reliability and precision of the method was performed using certified reference materials (1000 µg/mL, 2% HNO_3_; High-Purity Standards, USA). The sample (0.5 g) was digested with 3 mL of nitric acid (HNO_3_; 65%, P.A., ACS, ISO; PanReac AppliChem, Spain) in a porcelain crucible, heated for 30 min at 150 ± 5°C until the formation of brown vapor, then 2 mL of perchloric acid (HClO_4_; 70%, P.A., ACS, ISO; PanReac AppliChem, Spain) at 200 ± 5°C until the solution became translucent and white vapor formed, indicating complete digestion of the organic matter. Finally, 3 mL of hydrochloric acid (HCl; 37%, ACS, ISO; PanReac AppliChem, Spain) was added without heating and allowed to cool to room temperature, the digested sample was transferred to 50 mL volumetric flask. The transfer was performed using qualitative filter paper (3hw, 110 mm, 65 g m^-2^; Boeco, Germany) and the volume of the solution was brought to 50 mL with deionized water. With the information obtained from the calibration curves prepared with standards of each mineral, the data for each element were expressed in milligrams per kilogram of dry sample (mg kg^−1^).

### 2.4. Data analysis

A cluster analysis was performed to obtain different typologies of bean materials based on the information of the different variables related to nutritional composition. The number of clusters was determined by multivariate analysis of variance, where the difference between the typologies was determined using the Hotelling test [47]. The typologies were compared by means of a permutation test (PERMANOVA) that evaluates whether the multivariate means (centroids) of groups differ on an array of distances using the *adonis2* function of the *vegan* package. Subsequently, an analysis of variance was performed using linear mixed models (LMM), using a DGC (Di Rienzo, Guzman, and Casanoves) test (P < 0.05) to determine the differences between typologies. Typology was adjusted as a fixed factor and genotype as a random factor within the LMM. The DGC test [48] was used because it produces statistically homogeneous groups of means that do not overlap, facilitating the interpretation of results. Additionally, DGC maintains a Type I error rate per experiment lower than the nominal α level (0.05), standing out by showing the lowest Type II error rates among all methods. The above having greater power than traditional tests and other methods based on clusters, being particularly efficient when the number of treatments is large. A principal component analysis (PCA) was also performed to visualize each of the variables tested that were related to each of the bean typologies found, as well as a Monte Carlo test to determine the variance explained by the typologies. A nutritional composition index (NCI) was created to identify nutritionally superior bean genotypes [49]. The data processing carried out to obtain the NCI value corresponds to identifying the variables that have a positive and negative impact on the nutritional value. First, outliers were removed from each variable using the Interquartile Range (IQR). The value of each variable is transformed between a range of 0 (minimum) to 1 (maximum) under the criteria of: i) more is better, suitable for standardizing the scores of the properties of the bean genotypes that were associated with values close to one (1); and ii) less is better, those characteristics whose values were close to zero (0), an approach that has been used in other studies [49]. The general equation for the NCI can be expressed as follows:

For variables where “*more is better*”:


$$\begin{array}{c} X_{normalized}=\frac{X-X_{min}}{X_{max}-X_{min}} \end{array}$$

For variables where “*less is better*”:


$$\begin{array}{c} X_{normalized}=\frac{X_{max}-X}{X_{max}-X_{min}} \end{array}$$

Where:

X is the value of the variable to be normalized

$X_{max}$ is the maximum value of the variable in the dataset

$X_{min}$ is the minimum value of the variable in the dataset

$X_{normalized}$ is the transformed value between 0 and 1

The final NCI can be calculated as the average of all normalized variables:


$$\begin{array}{c} NCI=\frac{\text{1}}{n}\sum_{i=\text{1}}^{n}{X_{i}}_{normalized} \end{array}$$

Where:

*n* is the total number of variables considered

${X_{i}}_{normalized}$ is each normalized variable

After that, a Pearson correlation analysis was performed between the different variables, and only the results that presented a statistically significant correlation were plotted (P < 0.05). The functions *fviz_dend* and *fviz_pca_ind* of the “factoextra” package were used to perform the cluster analysis as well as the PCA. The LMM were made using the *lme* function in the *nlme* package, “*ggplot2*”, “*factoextra*” and “*corrplot*” in the R language software, version 4.4.1 [50].

## 3. Results

**Fig 1:**
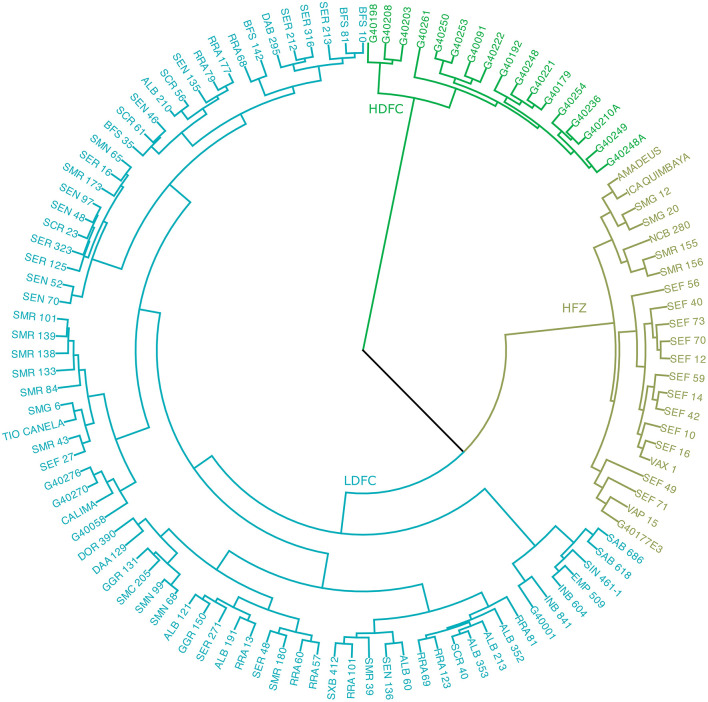
Observed typologies of 112 bean genotypes evaluated based on nutritional composition of seeds (Ward method, Euclidean distance): High Fe and Zn (HFZ) typology; high dietary fiber and cellulose (HDFC) typology; and low dietary fiber and cellulose (LDFC) typology.

According to the results obtained from the cluster analysis, which was based on the grouping of the means of each of the variables evaluated in the 112 accessions and advanced lines of beans, three typologies were found (Fig 1): 1. High Fe and Zn (HFZ), 2. High dietary fiber and cellulose (HDFC) and 3. Low dietary fiber and cellulose (LDFC) which explain ~28.6% of the variation and the differences between conglomerates are statistically significant according to the permutation test. Typology HDFC was found to be the most distant from the others, mainly due to its high fiber and cellulose content, primarily related to accessions of *P. acutifolius*.

**Fig 2:**
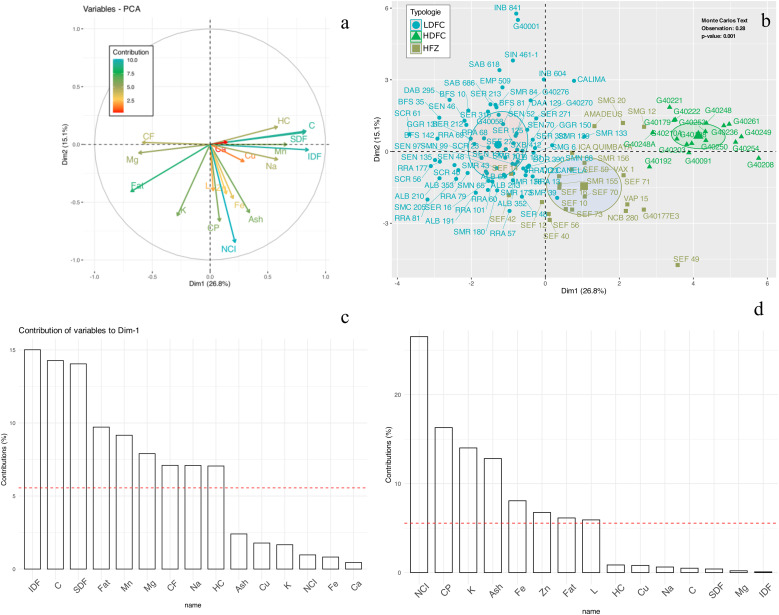
Principal component analysis (PCA) projection of variables related to nutritional composition of seeds of 112 bean genotypes: (a) correlation circle between the different variables of nutritional composition; (b) three different typologies of bean genotypes; and (c) and (d) contribution of variables related to nutritional composition of the principal components PC1/PC2 of PCA under different typologies of bean genotypes, the variables with values above the red line contributed significantly in each of the components. The gradient from blue to red indicates a higher to lower level of contribution of the variance explained by each variable in the components, respectively. High Fe and Zn (HFZ); high dietary fiber and cellulose (HDFC); and low dietary fiber and cellulose (LDFC). IDF: Insoluble dietary fiber; C: cellulose; SDF: soluble dietary fiber; Mn: manganese; Mg: magnesium; CF: crude fiber; Na: sodium; HC: hemicellulose; Cu: copper; K: potassium; NCI: nutritional composition index; Fe: iron; Ca: calcium; CP: crude protein; Zn: zinc; and L: lignin.

Of the explainable variance, 41.9% was observed using principal component analysis (PCA, Fig 2a) where PC1 explained 26.8% variance, opposing bean genotypes with high insoluble dietary fiber (IDF), soluble dietary fiber (SDF) and cellulose (C) values with those genotypes that presented high fat levels (Fig 2b and 2c). PC2 explained 15.1% variance, opposing bean genotypes with high nutritional composition index (NCI) as well as high CP, ash and K concentrations (Fig 2b and 2d). The NCI synthesizes the nutritional value based on nutritional composition of the seed. These typologies in the PCA explained 28% of the variance, based on the results of the Monte-Carlo test (Fig 2b). The nutritional composition contributed significantly to each of the two principal components, which are shown in Fig 2c and 2d, respectively.

Table 1 shows the means of each of the variables related to the nutritional composition of the three typologies that were formed with the 112 bean genotypes. It was found that crude protein (CP), lignin (L) and calcium (Ca) levels did not present differences among the typologies (P > 0.05), however, variables such as SFD, IDF, C and manganese (Mn) were different among the three typologies (P < 0.05). The characteristics of each of the three typologies formed are described below.

**Low dietary fiber and cellulose (LDFC; n = 73, 65.1% of the total genotypes):** The bean genotypes of this typology are characterized by having the lower values of SDF, IDF, C and Mn; traits that make them totally different from the other two bean typologies (Table 2). Likewise, variables such as hemicellulose (HC), sodium (Na) and copper (Cu) were found to be different from the other two typologies. However, these variables were not different between HDFC and HFZ typologies. The LDFC typology presented a lower value of the nutritional composition index (NCI) of 0.33. The genotypes SEN 135, G 40058 and SAB 686 showed lower values of SDF, IDF and C, respectively.

**Table 2:** Nutritional composition and nutritional composition index (NCI) of seeds of 112 bean genotypes grouped into three different typologies: high Fe and Zn (HFZ); high dietary fiber and cellulose (HDFC); and low dietary fiber and cellulose (LDFC).

Variable	Acronym	LDFC	HDFC	HFZ	overall average	P-value
Ash	Ash	3.93±0.05^b^	4.38±0.1^a^	4.13±0.07^b^	4.04±0.04	0.0003
Crude protein	CP	18.58±0.07	18.79±0	18.78±0.04	18.65±0.05	
Fat content	Fat	1.22±0.02^a^	0.76±0^b^	1.27±0.05^a^	1.16±0.02	<0.0001
Soluble dietary fiber	SDF	16.77±0.41^c^	25.64±0.5^a^	22.11±0.74^b^	19.17±0.45	<0.0001
Insoluble dietary fiber	IDF	7.17±0.09^c^	11.85±0.3^a^	8.05±0.35^b^	8.05±0.19	<0.0001
Hemicellulose	HC	9.6±0.41^b^	13.79±0.5^a^	14.06±0.72^a^	11.11±0.37	<0.0001
Cellulose	C	5.42±0.11^c^	10.2±0.3^a^	6.15±0.31^b^	6.29±0.19	<0.0001
Lignin	L	1.75±0.1	1.65±0.1	1.9±0.19	1.76±0.08	
Crude fiber	CF	6.23±0.23^a^	3.45±0.2^b^	5.9±0.34^a^	5.74±0.19	<0.0001
Calcium	Ca	120.87±3.23	123.69±8.5	123.02±3.92	121.72±2.57	
Magnesium	Mg	88.71±2.2^a^	59.27±2.3^b^	62.11±2.19^b^	79.02±1.98	<0.0001
Sodium	Na	54.15±3.23^b^	93.5±6.8^a^	95.29±6.63^a^	68.2±3.22	<0.0001
Potassium	K	567.04±9.71^a^	485.27±16^b^	589.85±22.2^a^	559.11±8.55	0.0005
Copper	Cu	2.62±0.06^b^	2.89±0.1^a^	3.05±0.15^a^	2.75±0.05	0.0027
Iron	Fe	30.18±0.85^b^	34.31±3.4^b^	39.98±1.66^a^	32.73±0.89	<0.0001
Zinc	Zn	8.62±0.16^b^	8.27±0.3^b^	10.95±0.61^a^	9.02±0.19	<0.0001
Manganese	Mn	3.9±0.14^c^	6.29±0.3^a^	4.6±0.21^b^	4.4±0.13	<0.0001
Nutritional Composition Index	NCI	0.33±0.02^a^	0.35±0^a^	0.53±0.03^b^	0.37±0.01	<0.0001

Mean ± Standard error. ^a, b, c:^ Averages with a letter in common between columns are not significantly different at 5% probability.

**High dietary fiber and cellulose (HDFC; n = 17, 15.1% of the total genotypes):** This typology is characterized by having higher values of the variables SDF, IDF, C and Mn (Table 2). Likewise, this typology differs from the other two typologies in terms of values of ash, fat, CF, and K. It should be noted that this typology is only composed of *Phaseolus acutifolius* germplasm accessions such as G 40253, G 40254, G 40250, G 40249 (which were specifically collected between Ciudad Juárez and Ciudad de Chihuahua, Mexico), among others (Fig 1).

**High Fe and Zn content (HFZ; n = 22, 19.8% of the total genotypes):** The main nutritional characteristics of the genotypes that make up this typology were higher values of Fe, Zn, CP and ash levels with average values of SDF, IDF, C and Mn (Table 2). This resulted in a higher average value of NCI of 0.53. Four bean genotypes SEF 56, SEF 10, SEF 70 and SEF 12 stood out in this typology, with an average Fe content of 62.7, 51.8, 49.4 and 45.8 mg kg^−1^, respectively. Of note in this typology is G 40177E3, a wild accession of *Phaseolus acutifolius*, which presented a content of 42.1 mg kg^−1^ of Fe and 17.1 mg kg^−1^ of Zn. Additionally, *P. vulgaris* lines SEF 49 and SEF 10 presented higher values of NCI. These two lines with red seed color are of Mesoamerican origin and were generated from an interspecific cross of *P. vulgaris* × *P. coccineus* × *P. acutifolius*, as well as a *P. vulgaris* line. Two other bean lines highlighted include NCB 280 and the line VAP 15 that was also generated from the interspecific cross of *P. vulgaris* × *P. acutifolius* × *P. parvifolius* (Fig 1).

**Fig 3:**
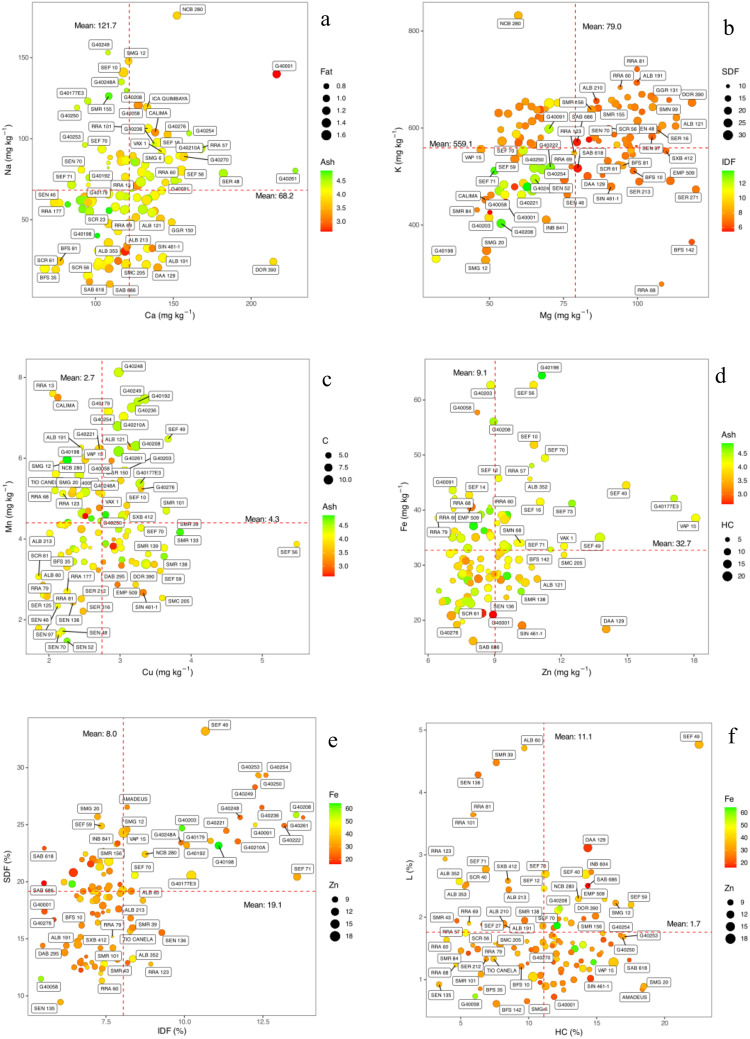
Relationships between different variables of nutritional composition of seeds of 112 bean genotypes as a function of the gradient (change in the color gradient of green to red indicates from higher to lower value) and the magnitude (size of the circle) of the iron (Fe) and Zn (Zn) parameters that are related to the different variables on the Y and X axes. The red dotted lines on the axes correspond to the means of the dependent and independent variables. 3a: sodium (Na) and calcium (Ca) concentration as a function of ash (Ash) and fat content (Fat). 3b: potassium (K) and magnesium (Mg) concentration as a function of insoluble dietary fiber (IDF) and soluble dietary fiber (SDF). 3c: concentration of manganese (Mn) and copper (Cu) as a function of ash and cellulose (C) level. 3d: concentration of iron (Fe) and zinc (Zn) as a function of ash and hemicellulose (HC) level. 3e: insoluble dietary fiber (IDF) and soluble dietary fiber (SDF) as a function of iron (Fe) and zinc (Zn) concentration. 3f: lignin (L) and hemicellulose (HC) level as a function of iron (Fe) and zinc (Zn) concentration.

Fig 3 shows the relationships of different variables related to the nutritional composition of seeds of 112 bean genotypes. For example, genotypes such as G 40001, G 40261 (*P. acutifolius*) and DOR 390 (*P. vulgaris*) presented higher levels of Ca. In the case of seed Na levels, genotypes such as NCB 280 (*P. vulgaris*), SMG 12 (*P. vulgaris × P. coccineus × P. acutifolius × P. dumosus*), G 40249 (*P. acutifolius*) and SEF 10 (*P. vulgaris × P. coccineus × P. acutifolius*) presented higher values, the latter having the highest fat content (Fig 3a). For the trend between seed Mg and K levels (Fig 3b), it was found that genotypes such as DOR 390, SER 271 (*P. vulgaris*) and BFS 142 (*P. vulgaris× P. coccineus*) presented values higher than 118 mg kg^−1^ of Mg and for the case of K the genotype NCB 280 (*P. vulgaris*) presented the highest value of 800 mg kg^−1^. It was observed that higher Mg contents were related to a lower IDF value (higher red color gradient, Fig 3b). SEF 56 (*P. vulgaris × P. coccineus × P. acutifolius*) was found to have higher Cu level (Fig 3c), distinct from the other genotypes evaluated. On the other hand, genotypes with higher cellulose magnitude (C, circle size) had a high Mn level (Fig 3c), where genotype G 40248 (*P. acutifolius*) was found to be enriched with this mineral.

Regarding the most important mineral levels in beans, Fig 3d shows the trend for Zn and Fe in relation to the amount of ash and hemicellulose. It was found that genotypes such as VAP 15, a product of an interspecific cross of *P. vulgaris × P. acutifolius × P. parvifolius*, G 40177E3 (*P. acutifolius*) and SEF 40 (*P. vulgaris × P. coccineus × P. acutifolius*) presented Zn concentrations higher than 15 mg kg^−1^. As for the seed Fe concentration, different genotypes of *P. acutifolius* (G 40198, G 40203, G 40058, G 40206) and those derived from a triple cross of *P. vulgaris × P. coccineus × P. acutifolius* (SEF 56, SEF 10, SEF 70) showed contents higher than 50 mg kg^−1^. Likewise, it was found that both Zn and Fe and hemicellulose values were high (Fig 3d). Finally, as a function of the seed Fe and Zn gradient, the relationship between SDF and IDF showed superior values in *P. acutifolius* accessions (Fig 3e) while the relationship between L and HC (Fig 3d) showed outstanding values in the genotype SEF 49.

**Fig 4:**
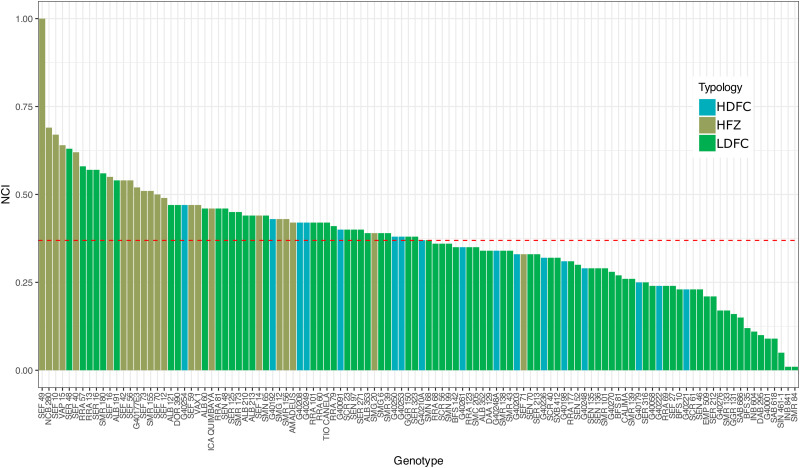
Nutritional composition index (NCI) differences in seeds of 112 bean genotypes of three typologies: high Fe and Zn content (HFZ); high dietary fiber and cellulose (HDFC); and low dietary fiber and cellulose (LDFC). Red line shows the average value of NCI (0.37).

Fig 4 shows the nutritional composition index (NCI) value of each bean genotype in each of three typologies (HDFC, HFZ, LDFC). The NCI is a dimensionless relative value that indicates which is the best line or accession from the absolute values measured with nutritional composition (ash, fiber, protein, among others) as well as with variables related to mineral status (Fe, Zn, Ca, Mg, Cu, among others). The NCI was based on converting the values of each variable into scores with a range between values from 0 to 1, using a standardized scoring function, under the criteria: i. More is better, suitable for standardizing the values of each of the variables that have a positive impact on nutrition associated with values close to one (1); ii. Less is better, for those variables that have a negative impact on nutrition such as copper (Cu), and insoluble dietary fiber (IDF) whose values were close to zero (0). It was found that out of the total 112 genotypes, 58 had a value equal to or greater than 0.37 and only five genotypes (SEF 49, NCB 280, SEF 10, VAP 15, SEF 40) of the HFZ typology and one (SER 48) of the LDFC typology, had an NCI value greater than 0.6 (Fig 4). SEF 49 represented the maximum NCI value of 1.0 compared to the other genotypes in the typology of HFZ. It was found that genotypes such as G 40001 of the *Phaseolus acutifolius* presented a lower NCI value belonging to the LDFC typology.

**Fig 5:**
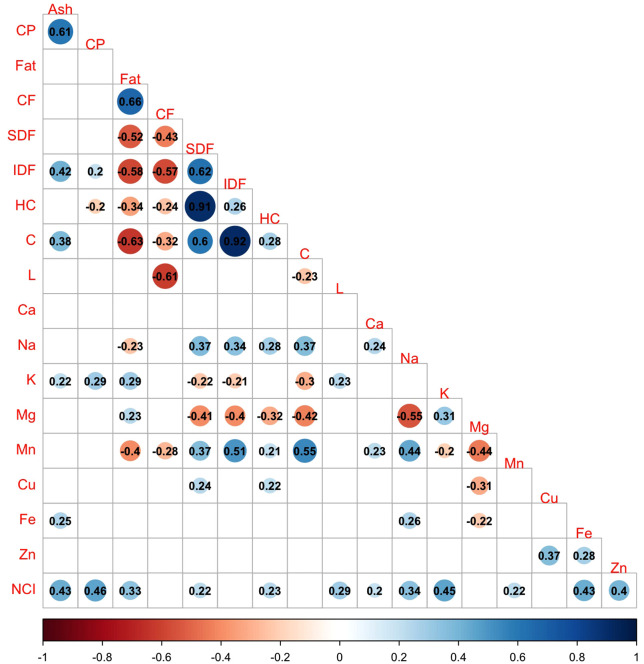
IDF: Insoluble dietary fiber; SDF: soluble dietary fiber; Fe: iron; K: potassium; Na: sodium; Ca: calcium; Mg: magnesium; Mn: manganese; Cu: copper; Zn: zinc; CP: crude protein; CF: crude fiber; NCI: nutritional composition index; HC: hemicellulose; L: lignin and C: cellulose. The numbers shown presented significant correlations (P < 0.05) in the color gradient from red to blue showing negative and positive correlation, respectively.

When analyzing the relationship between the different variables, it was found that NCI did not correlate with CF, IDF, C, Mg and Cu (P > 0.05), however, with the other variables there was a positive correlation (P < 0.05). Among the variables that showed greater correlation with NCI were CP, K, Fe, and Zn (Fig 5). In the case of Fe content, there was a positive correlation with Ash and Na, but a negative correlation with Mg. Likewise, Zn content correlated with both Cu and Fe (Fig 5). The variables with greater negative correlations with the other variables were Fat, SDF, IDF, HC, and C (Fig 5).

**Fig 6:**
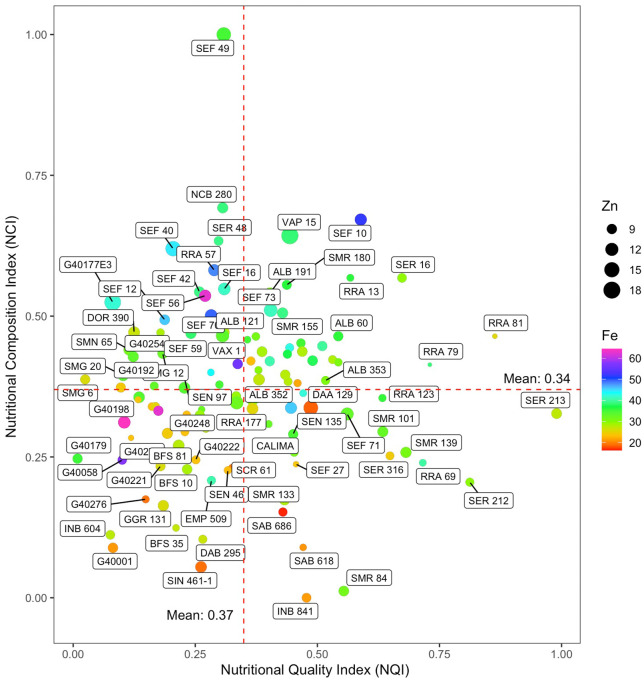
Relationship between different indices that consolidate different variables of nutritional composition, bioactive compounds and antioxidant activity in seeds of 112 bean genotypes as a function of the gradient (change of purple and red color from higher to lower value) and the magnitude (size of the circle) of the iron (Fe) and zinc (Zn) parameters. The dashed red lines on the axes correspond to the means of the dependent and independent variables.

When relating the Nutritional Composition Index (NCI) and Nutritional Quality Index (NQI) [21] it was found that SEF 10, SER 16 and RRA 13 had the most similar values between the two indexes, possessing an adequate nutritional composition, mineral status, bioactive compounds and antioxidant activity, contrary to what was found for genotypes such as G 40001 and INB 604, which presented lower values in terms of both NCI and NQI (Fig 6). Importantly, in the present study, SEF 49, NBC 280, VAP 15, and SEF 10 exhibited higher values of nutritional composition and mineral status while materials such as SER 213, SER 212, and RRA 81 stood out for their superiority in terms of bioactive compounds and antioxidant capacity (Fig 6, [21]).

## 4. Discussion

This study enabled the identification of distinct bean genotype typologies based on seed nutritional composition, and the selection of nutritionally superior lines using the Nutritional Composition Index (NCI). Regarding mineral composition, the high Fe and Zn (HFZ) typology with an average value of 0.53 in the NCI, comprises genotypes developed from interspecific crosses of *P. vulgaris × P. coccineus × P. acutifolius* (SEF 49 and SEF 10), as well as a *P. vulgaris* genotype (NCB 280) and the VAP 15 line from a triple cross of *P. vulgaris × P. acutifolius × P. parvifolius*. In terms of fiber content, the high dietary fiber and cellulose content (HDFC) typology includes mainly genotypes of the species *P. acutifolius* (G 40250, G 40253, G 40254) with moderate range of NCI values that were higher than 0.3. It was found that germplasm accessions such as G 40001 of the species *P. acutifolius* presented markedly lower NCI value within the group of genotypes belonging to LDFC typology.

A high NCI value indicates that seeds contain greater concentration of essential nutrients (protein, Fe, Zn, K, fiber, etc.), but translating this into real dietary benefits requires considering bioavailability and absorption —i.e., the fraction of those nutrients the body can digest, absorb, and metabolize [51] Thus, a high NCI values reflects a larger nutrient reserve per grain (more “supply” per serving) [12,52] and increases dietary potential, but the actual benefit depends on how much of that content is accessible to the organism. Bioavailability and absorption are affected by antinutrients [53]: tannins and phytates can reduce Fe, Zn, and other mineral absorption, so a genotype with high seed Fe but also with high phytate may provide less absorbable iron. In addition, a high proportion of insoluble fiber can limit mineral release during digestion [54].

### 4.1. Nutritional composition and nutritional typologies

Within the nutritional profile of the bean genotypes evaluated, SEF 49, SEF 10, NCB 280 and VAP 15, belonging to the typology (HFZ) stand out for their significant contribution with an average value of 18.78% in crude protein, being an essential nutritional component for normal growth and development of the human body [2]. It is important to keep in mind that genetic factors influence protein concentration in bean varieties [1,55]. In addition, genotype-environment interactions and cultivation practices [2,56–58] could also influence protein quantity and quality [59,60]. Acid soil and high temperature stress affect nutritional quality of common bean genotypes [21]. The genotypes evaluated in the southern region of the Colombian Amazon show promising protein potential, serving as a staple food to supplement the human diet, with seed protein contributions higher than 17.3% [16,61]. The crude protein content of different varieties of beans grown and consumed in the state of Zacatecas, Mexico [62], reported values ranging from 19.2 to 26.8%, with the Reatan variety having the highest content, significantly exceeding the bean materials that were evaluated in this study under acid soil and high temperature stress conditions.

Lipids present in beans consist mainly of fatty acids [1,63]. However, it was observed that this lipid content in the three typologies identified in the study (HDFC, HFZ and LDFC) is low and uniform among the different genotypes, regardless of their origin. This finding is in agreement with previous reports of Redondo-Cuenca et al. [59] and Alvarado-López et al. [58], who also highlight that, due to their low content and the presence of polyunsaturated fatty acids, these beans offer benefits to human health by reducing the risk of cardiovascular diseases, therefore, this food becomes an important part of a balanced and healthy diet [16,64]. On the other hand, high levels of ash have been observed in the genotypes analyzed, with a mineral content close to 4.6% [65,66], being notably higher in the HDFC typology (4.38%), which comprises genotypes of the species *P. acutifolius*. The variability in ash content can be attributed to the genetic heritability of each material [58], suggesting that these genotypes provide valuable and essential minerals for optimal body development [67,68].

According to Mecha et al., [65] crude fiber content in bean genotypes, both local and improved, can vary from 5.6 to 10.8%. This variability coincides with the values found in different genetic materials, indicating the presence of a significant amount of fiber in the seeds [68]. In general, *P. acutifolius* germplasm accessions such as G 40253, G 40254, G 40250 and G 40249 categorized with the HDFC typology, provide higher amounts of dietary fiber to the digestive system. This is mainly due to the intrinsic characteristics of the bean cultivars [2,67]. The contribution of dietary fiber provides a vital substrate for the development of beneficial intestinal microbiota and helps prevent the development of pathogenic microorganisms, generating an anti-inflammatory and protective effect against various diseases of the colon, such as ulcerative colitis and colon cancer [69]. Soluble dietary fiber (SDF) influences lipid metabolism in the body, while insoluble dietary fiber (IDF) increases stool weight and volume, accelerating intestinal transit [70]. All forms of fiber, except for lignin, such as cellulose and hemicellulose, are fermented by intestinal bacteria, with a fermentation capacity ranging from 20 to 90% [71]. This fact suggests that a higher intake of these substances, derived from adequate food sources, contributes to maintaining the health of the gastrointestinal tract [68,69].

Regarding the mineral composition of beans, research has shown that they are an excellent source of macro- and micronutrients and have higher levels of minerals compared to other legumes [1,72]. For example, in the LDFC typology, low values of Mn, Na and Cu were recorded, and a higher concentration of Mg, especially in genotypes such as SEN 135, G 40058 and SAB 686. The HDFC typology is characterized by higher values of Mn and K, especially in accessions belonging to the species *P. acutifolius*, with an average value of 6.9 and 485.27 mg kg^−1^, respectively. On the other hand, in the HFZ typology, high concentrations of Fe (39.98 mg kg^−1^) and Zn (10.95 mg kg^−1^) were recorded, and in lower proportion of K (589.85 mg kg^−1^) and Na (95.29 mg kg^−1^), mainly in lines with red seed color (SEF), obtained through an interspecific cross of *P. vulgaris × P. coccineus × P. acutifolius*.

The increase of Fe and Zn content in seeds of common bean (biofortification) has been the object of bean breeding programs [73–75]. The HarvestPlus program has established an iron concentration of 90–94 mg kg^−1^ for materials defined as biofortified beans [76–78]. When compared to the concentrations of the materials evaluated in the present study, none reached to this threshold range. However, SEF 56 had an Fe concentration of 62.7 mg kg^−1^, while accessions G 40198 and G 40203 showed moderate values of 64.4 and 62.6 mg kg^−1^, respectively. However, recent studies show results of increased biofortification capacity, for example Suárez et al. [14], in seeds of advanced bean lines of F_4_ and F_5_, found a mean Fe concentration of 82 ± 2.6 mg kg^−1^ and 65.6 ± 4.7 mg kg^−1^, respectively. Likewise, Lamptey et al. [79] using six generations of two populations made from crosses between pairs of low Fe and low Zn; and high Fe and moderate Zn genotypes (respectively Cal 96 *×* RWR 2154; MCR-ISD-672 *×* RWR 2154), found Fe concentrations ranging from 60.7 to 101.6 mg kg^−1^. On the other hand, Fe concentrations were found between 54–100 mg kg^−1^ in seeds of genotypes from different biparental populations using Cerinza × G 10022 [80], and between 28–95 mg kg^−1^ of Fe in populations of G 21242 × G 21078 [81], and between 35–97 mg kg^−1^ of Fe in populations of G 14519 × G 4825 [82]. Fernandez and Sanchez [83] reported that the Fe levels found in the varieties used showed that Flor de mayo bean presented the maximum Fe content, while Frijol Bayo had the minimum content, their concentrations being 109.8 and 72.8 mg kg^−1^, respectively. It has been reported that the range of variation in Fe concentration is caused by the strong effects of genotype, environment and genotype × environment depending on the growing environment [74]. These advances in the development of biofortified varieties of common bean (*Phaseolus vulgaris* L.) could address acute micronutrient deficiencies with consequent improvement in nutrition and health of women, children, and adults in different regions of the world [79].

Variability in mineral uptake in bean seeds is attributed to the genetic composition of each plant material and its color characteristics [2,58]. However, the nutritional quality characteristics of the same plant material can differ significantly when grown under different environmental conditions [84]. Therefore, climatic patterns also influence nutrient uptake by affecting their solubility and availability in the root zone [59,74]. According to research conducted by Ejigu et al. [74] and Philipo et al. [85], genotype, environment and genotype-environment interaction have been found to be responsible for significant variations in bean Fe and Zn concentrations, and to a lesser extent in Mg, K, Cu and Mn concentrations in seed [86,87]. These findings suggest that climatic factors such as temperature and precipitation, biotic factors such as diseases and pests, and soil factors such as acidity and nutritional availability could influence nutritional composition of seeds, and the selection of genotypes adapted to different agroclimatic conditions [74,88].

### 4.2. Nutritional composition index and identification of nutritionally superior bean genotypes

The nutritional composition index (NCI) developed in this study is based on the same mathematical principle as the nutritional quality index (NQI) presented by Suárez et al. [21] using the same 112 bean materials. However, the NQI was focused on identifying bean materials with high content of bioactive compounds and antioxidant activity. This study by Suárez et al. [21] identified five groups of bean materials: i. highly bioactive and functional (HBF), ii. moderately bioactive and functional (MBF), iii. moderate antioxidant content with influence of pigments (MACP), iv. moderately antinutritional with limited antioxidant potential (MALAP), and v. antinutritional, low bioactive and functional (ALBF) in which three interspecific genetic lines were identified (SER 212, SER 213 and RRA 81), with NQI values above 0.8.

Use of NCI and NQI [21] in bean breeding programs will allow the identification of bean materials that can serve as useful parents to develop nutritionally superior and stress resistant beans. This has been of high interest recently [75,89] and is part of the research focus of breeding programs that have grouped bean materials based on phenotypes related to chemical composition and mineral content, identifying materials with higher nutritional value [15,74,85,90]. Among the bean cultivars evaluated, genotypes with high concentrations of one or more micronutrients have been identified based on NCI values, valuing the high nutritional composition of materials such as SEF 49, SEF 10 and SEF 40, which come from the cross between [(ALB 74 × INB 841)F_1_ × RCB 593] (*P. coccineas* × *P. acutifolius* × *P. vulgaris*). These materials in addition to having traits related to the ability to adapt to drought and high temperature stress conditions [91,92], have shown adaptation to the combined stress of acid soil and high temperatures [19–21] and a moderately bioactive and functional content specifically with low and moderate concentration of condensed tannins and carotenoids as well as high antioxidant activity [21] together with high levels of crude protein, fat, soluble dietary fiber and a moderate level of K and Zn. These SEF materials presented an average of 43.7 mg kg^−1^, where SEF 10 presented the highest Fe content of 51.9 mg kg^−1^. Likewise, we highlight the material NCB 280 [(SXB 123 × DOR 677) × SEN 34] (*P. vulgaris*) which has shown to have a high yield under drought conditions [93] as well as adaptation to combined acid soil stress and high temperature [20] whose main nutritional trait is to have higher levels of K (831.8 mg kg^−1^) and Na (175.9 mg kg^−1^), with a moderate level of Fe (38.8 mg kg^−1^) in the grain. However, this material presented a very low NQI due to its high content of antinutritional compounds, classifying it as “moderately antinutritional with limited antioxidant potential” [21]. Another material that presents a high NCI value is VAP 15, which also has a very desirable characteristic for bean breeding in the future due to its ability to facilitate hybridization of common bean and tepary bean without the need for embryo rescue [23], and this line resulted from the cross between three bean species *P. vulgaris, P. acutifolius* and *P. parvifolius* [((INB 834 × G 40264)F_1_ × INB 841)F_1_ × SMR 139]. In the present study, this material had a high crude protein level of 18.9% and a high Zn concentration of 18 mg kg^−1^, and these two variables being the highest values among the 112 materials evaluated, in addition to a high flavonoid content and a high antioxidant capacity, classifying it as “moderately bioactive and functional content” [21]. Finally, SER 48 [(SXB 123 × EAP 9653-16B-1)F_1_ × SXB 125] had a high level of minerals such as Ca, Mg and K and a high level of ash and crude protein.

Increasing Fe and Zn concentrations and other desirable traits in bean seeds, through access and use of genetic diversity and adaptive processes, drives the development of biofortified bean lines [73,88], added to the bioavailability of iron in the common bean as an additional factor to consider in terms of human nutrition. These concentrations of seed Fe and Zn are affected, in addition to genetic variability, by different factors related to soil acidity and fertility, such as the availability of water and nutrients in the soil, as well as ambient temperature. For example, it was recently reported that the absorption capacity of Zn and Fe concentration in seeds was low in soils with pH level equal to 5.3 compared to the optimum soil pH (6.5), however, materials such as NUA 11 and NUA 17 showed a high performance in Zn and Fe absorption at pH 5.3 [94]. When comparing these results, we observed that the average values of both Fe and Zn are higher than those presented in the present study even at the same soil pH levels. These possible differences in Fe and Zn levels are because of genotype, since the data reported by Magomere et al. [94] corresponds to the selection of large seeded Andean bush materials with a higher mineral concentration and a type of speckled red seed which were named as NUA (Nutritional Andean) lines [81]. At a low soil pH of 5.3, the uptake capacity of Zn and Fe in both leaves and seeds was low compared to the optimum soil pH of 6.5. However, some genotypes such as NUA 11 and NUA 17 showed high Zn and Fe uptake performance at pH 5.3. Soil acidity is a factor that affects nutrient accumulation, for which activities have been carried out to improve pH such as liming the soil, but it has been reported that this process had no effect on the Fe concentration in seed of three bean genotypes (Voyager, T39, UI911) [95]. Regarding P status in soil, it has been reported that seed Fe has a medium relationship with available P (r = 0.54, P < 0.001) [96], likewise other studies have reported a high correlation with seed Fe with available P in soil between 0.84 to 0.89 (P < 0.001) [85,97]. As for seed Zn concentration, a medium correlation with soil P availability has been reported (r = 0.68, P < 0.001), but this depends on the location [96]. However, Philipo et al. [85] reported a negative correlation between seed Zn and soil available P (r = −0.59, P < 0.001). Other studies have shown that the application of high P to the soil significantly increased the yield and seed P of cowpea, but not the seed Zn [98]; a trend that has also been reported for common bean [99].

Likewise, it has been reported that under drought conditions nutrient concentration within the seed is not discernably impacted [100]. This study detected genotypic differences in seed Fe, for example NCB 226 and CARIOCA maintained higher concentrations of Fe in the seeds under irrigated conditions, while SEN 56 and CARIOCA showed higher concentrations of Fe in the seeds under drought conditions. On the other hand, it was revealed that under drought stress conditions Fe levels in bean seeds decrease while Zn, lead, protein and phytic acid levels increase [101]. Another study reported that the seed Fe was more affected by drought stress and was reduced at all phenological stages, however, when comparing between bean genotypes, the seed Fe of white bean was lower than that of the red and Chitti genotypes [102]. Diaz et al. [39] analyzed the effect of drought stress on seed Fe (SdFe) and seed Zn (SdZn) concentrations in three Mesoamerican biparental populations: (1) SCR 16 × SMC 40, (2) SMC 33 × SCR 16, and (3) SMC 44 × SCR 9 that were obtained from five elite lines developed in the CIAT bean breeding program. Although they found no effect of drought stress on SdFe and SdZn in the populations evaluated in all trials, the SMC parental lines (SMC 40, SMC 44, and SMC 33) performed better in terms of SdFe and SdZn than the SCR parental lines (SCR 9 and SCR 16) [39]. In general, the study of seed Fe and Zn levels shows significant differences between lines evaluated under drought stress (DS) and well-watered (WW) field conditions. For example, Smith et al. [103] did not detect linear variation in seed Fe and seed Zn among twelve dry bean lines in DS and WW environments, however, other studies have reported a wide genetic variation in WW conditions [97,104–106].

The Nutritional Composition Index (NCI) is important because it synthesizes into a single indicator the basic seed composition characteristic that are key for food security and biofortification. These include a broad mineral profile (Fe, Zn, K, Mg, Ca, Mn, Cu); protein, fiber fractions (soluble, insoluble, cellulose, hemicellulose, lignin), ash, fat; and also incorporating directional nutritional criteria (for example, treating Na as a negative factor). By integrating variables with direct impacts on dietary supply, micronutrient concentrations bioavailability and physiological functions (gut health, glycemic response, mineral requirements), the NCI facilitates the prioritization of genotypes that improve the basic nutritional value of grains and guides selection decisions in bean breeding programs focused on improving traits of biofortification and food quality of bean materials grown under stress conditions. The Nutritional Quality Index (NQI) complements the NCI by evaluating distinct but equally relevant dimensions: bioactive and functional compounds (phenols, flavonoids, pigments, antioxidant activity) and the balance with antinutritional factors (tannins). While the NCI measures essential “nutrient concentrations”, the NQI measures the seed’s “functional capacity” and health-protective potential. Used together, NCI and NQI enable selection of bean lines that not only provide essential nutrients and target mineral concentrations (NCI) but also offer functional benefits associated with antioxidants and bioactive compounds or, conversely, present elevated levels of antinutrients that require management (NQI).

## 5. Conclusions

This study represents a significant advance in the characterization and evaluation of 112 bean genotypes cultivated under stress conditions in acidic soils and high temperatures in the western Amazon region of Colombia. The research highlights the development of the Nutritional Composition Index (NCI) as an innovative tool for evaluating and selecting bean genotypes with superior nutritional quality. The NCI, which considers multiple variables of nutritional composition and mineral status, provides a holistic view of the nutritional value of genotypes and serves as a potential selection index for breeding programs. The study identified three distinct nutritional typologies: high Fe and Zn content (HFZ), high dietary fiber and cellulose content (HDFC), and low dietary fiber and cellulose content (LDFC). Among the evaluated genotypes, those with high Fe and Zn content from the HFZ typology showed a mean NCI value of 0.53, particularly highlighting genotypes from interspecific crosses of *P. vulgaris* × *P. coccineus* × *P. acutifolius* (SEF 49 and SEF 10), as well as *P. vulgaris* genotypes, NCB 280 and VAP 15, developed from the interspecific cross of *P. vulgaris* × *P. coccineus* × *P. acutifolius*. Regarding fiber content, the HDFC typology mainly included germplasm accessions of P. acutifolius (G 40250, G 40253, G 40254) with moderate NCI values above 0.3. Among genotypes with low NCI values, the *P. acutifolius* germplasm accession (G 40001) presented the lowest value. This study also revealed significant correlations between nutritional variables, such as the positive relationship of Fe and Zn with ash and Na, and their negative correlation with Mg, providing new insights into how these factors interact and influence seed quality and nutritional value. This research not only provides an innovative tool like the NCI but also its use together with NQI to identify bean genotypes with superior nutritional value. This study contributes to the development of climate-resilient bean varieties that combine nutritional value with higher seed yields. Furthermore, the findings of this study also contribute to address critical food and nutritional security issues, particularly in regions with adverse agroclimatic conditions, such as Latin America and Eastern and Southern Africa.

## Supporting information

S1 File Variables monitored in the different materials. (XLSX)
